# Effect of different exercise modalities on nonalcoholic fatty liver disease: a systematic review and network meta-analysis

**DOI:** 10.1038/s41598-024-51470-4

**Published:** 2024-03-14

**Authors:** Yaqi Xue, Yang Peng, Litian Zhang, Yi Ba, Gang Jin, Ge Liu

**Affiliations:** 1 School of Economics and Management, Hebei University of Architecture, Hebei, China; 2 Beijing Normal University College of Physical Education and Sport, Beijing, China; 3 Qilu Normal University, Jinan, Shandong China; 4 Physical Education Department, Northeastern University, Shenyang, Liaoning China

**Keywords:** Health care, Health occupations

## Abstract

Physical exercise intervention can significantly improve the liver of patients with Non-alcoholic fatty liver disease (NAFLD), but it is unknown which exercise mode has the best effect on liver improvement in NAFLD patients. Therefore, we systematically evaluated the effect of exercise therapy on liver and blood index function of NAFLD patients through network meta-analysis (NMA). Through systematic retrieval of PubMed, Cochrane Library, Web of Science, EBSCO, and CNKI (National Knowledge Infrastructure), two reviewers independently screened the literature, extracted data, and assessed the risk of bias of the included studies by means of databases from inception to January 2023. The NMA was performed using the inconsistency model. A total of 43 studies, 2070 NAFLD patients were included: aerobic training (n = 779), resistance training (n = 159), high-intensity interval training (n = 160), aerobic training + resistance training (n = 96). The results indicate that aerobic training + resistance training could significantly improve serum total cholesterol (TC) (Surface under the cumulative ranking curve (SUCRA) = 71.7), triglyceride (TG) (SUCRA = 96.8), low-density lipoprotein cholesterol (LDL-C) (SUCRA = 86.1) in patients with NAFLD including triglycerides. Aerobic training is the best mode to improve ALT (SUCRA = 83.9) and high-density lipoprotein cholesterol (HDL-C) (SUCRA = 72.3). Resistance training is the best mode to improve aspartate transaminase (AST) (SUCRA = 81.7). Taking various benefits into account, we believe that the best modality of exercise for NAFLD patients is aerobic training + resistance training. In our current network meta-analysis, these exercise methods have different effects on the six indicators of NAFLD, which provides some reference for further formulating exercise prescription for NAFLD patients.

## Introduction

Non-alcoholic fatty liver disease (NAFLD) was originally defined in 1980^1^. It is defined as the presence of steatosis in more than 5% of hepatocytes, associated with metabolic risk factors (especially obesity and type 2 diabetes) and without excessive alcohol consumption (≥ 30 g/day in men and ≥ 20 g/day in women) or other chronic liver disease^2^. NAFLD is a major cause of cirrhosis and hepatocellular carcinoma. And it encompasses a spectrum of diseases ranging from steatosis with or without mild inflammation NAFLD to nonalcoholic steatohepatitis (NASH), which is characterized by necroinflammation and more rapid fibrosis progression than NAFLD^3^. Currently, NAFLD is considered one of the most common causes of chronic liver disease, causing 1.2 million deaths annually and rising to the eighth most common cause of death in the world^4^. In recent years, the prevalence of NAFLD has been increasing year by year in different regions of the world. According to a meta-analysis, the current global prevalence of NAFLD is an alarming 32.4%, which places a huge economic burden on society.^5^. In many parts of the world, NAFLD has become a more common chronic liver disease, and it is closely associated with obesity, type 2 diabetes mellitus (T2DM), dyslipidemia, and other patients^6^. For example, about 30.45% in South American states, Europe (23.71%), Korea (27.3%) and Japan (23–26%)^7–9^. As a result, complications of NAFLD place a significant health, economic, and patient experience burden on patients, their families, and society^10^. Because of the increasing prevalence of NAFLD, the search for an effective treatment has become an urgent issue.

NAFLD is a multi-system disease related to genetic, environmental and metabolic stress, including simple fatty liver disease, which progresses to NASH and cirrhosis (LC)^11^. The mechanism of NAFLD occurrence and development may be related to overproduction of reactive oxygen species (ROS) and oxygen species (OS). And it is also linked to DNA, lipid and protein oxidation and subsequent hepatocyte death^12^. Lipid metabolism disorder is also closely related to NAFLD, so Serum total cholesterol (TC), Triglyceride (TG), Low-Density Lipoprotein Cholesterol (LDL-C) and High-density Lipoprotein Cholesterol (HDL-C) in NAFLD patients show abnormalities^13^. In addition, Aspartate Transaminase (AST) and serum alanine aminotransferase (ALT) are good indicators for evaluating NAFLD patients^14^. However, NAFLD is the most prevalent liver disease worldwide and there is no approved pharmacotherapy. At present, pioglitazone and vitamin E are now recommended as effective drug therapy for NAFLD patients confirmed by biopsy^15^. Meanwhile diets high in caloric, high fat, and fructose-rich foods, along with a sedentary lifestyle, and obesity are significant risk factors implicated in the development and progression of NAFLD^16^. Therefore, making lifestyle changes may be a good choice. Exercise is a good way. It is an important factor affecting metabolism control by increasing physical activity of NAFLD patients. In some cross-sectional studies, it was found that NAFLD patients had low levels of physical activity^17,18^. Therefore, exercise intervention to improve the activity level of NAFLD patients is very important to cultivate a healthy lifestyle. The effect of exercise on NAFLD has been confirmed in previous studies^19,20^. Moderate intensity exercise of any degree is associated with a reduced risk of NAFLD and remission of NAFLD. The frequency of exercise plays a decisive role in reducing the incidence of NAFLD by 16% and improving the remission rate of NAFLD by 40%. Higher baseline exercise levels and increased weekly exercise over time were also associated with a reduced risk of NAFLD events and NAFLD resolution^21^. Exercise may be to reduce excessive ROS and OS production in NAFLD by regulating several mechanisms of action of exercise on NAFLD antioxidant enzymes and anti-inflammatory mediators. In addition, exercise on NAFLD patients is reflected in the effective improvement of TC, TG, LDL-C, HDL-C, AST and ALT levels^22^. This is very beneficial for the NAFLD patients.

Although previous systematic studies included different exercise interventions and indirect comparisons, it was not possible to determine the best exercise model^23,24^. Therefore, the effect of different exercise methods on NAFLD can be well determined through systematic review. In this study, we reviewed several randomized clinical trials (RCTs), discussed the efficacy of different exercise methods in NAFLD, and analyzed its characteristics using mesh meta-analysis. It aims to provide scientific and comprehensive reference for the formulation of exercise prescription for the treatment of NAFLD.

## Methods

Network meta-analysis is performed according to the preferred reporting items in the System Review and Meta-analysis (PRISMA) guide^25^. (PROSPERO: CRD42023457428).

### Literature search strategy

The literature search was performed for the related research studies, mainly from the following databases: PubMed, Cochrane Library, Web of Science, EBSCO, CNKI. The search keywords we used were (“Non-alcoholic fatty liver disease” OR “NAFLD” OR “non-alcoholic steatohepatitis” OR “NASH”) AND (“Randomized controlled trial” OR “Random” OR “RCT”) AND (“Exercise” OR “Training” OR “Aerobic training” OR Aerobic exercise OR “Resistance training” OR “Resistance exercise” OR “high-intensity interval training” OR “high-intensity interval exercise” OR “HIIT”). The meta-analysis is limited to January 2023, and the included study only includes randomized controlled trials.

### Inclusion and exclusion criteria

Studies were included according to the following criteria: (1) RCTs with exercises as the intervention treating patients with NAFLD; (2) Subjects were diagnosed as NAFLD through pathological or imaging examination; (3) There was no significant difference in the basic indicators of patients included before intervention; (4) Results indicators included triglyceride (TG), total cholesterol (TC), low-density lipoprotein (LDL-C), high-density lipoprotein (HDL-C), Alanine aminotransferase (ALT), and Aspartate aminotransferase (AST); (5) The data before and after intervention were obtained. Studies were excluded based on the following criteria: (1) Unable to obtain exact data; (2) The detection indicators did not meet the inclusion criteria; (3) Studies such as animal experiments, abstracts, case reports, reviews, systematic reviews, and repeated publications; (4) The mode, duration, and period of the movement are unclear.

### Data extraction

The two authors independently screened abstracts and full-text articles from these selected works, extracted and cross-checked the data. In case of disagreement, we consult a third party for mediation and reach consensus. In the literature screening process, the first thing is to read the title and abstract, and then the full text to determine the excluded literature. The following data were extracted from the selected works: research title; author name; publication time; sample size; interventions; type, intensity, frequency and duration of exercise; relevant patient outcomes (TC/TG/LDL-C/HDL-C/AST/ALT); and risks of literature bias.

### Quality assessment

The quality of the included studies was evaluated. Then two authors assessed the quality of the included studies. Any disagreement was discussed with a third reviewer. The two authors/It is important to use the Cochrane Handbook for Systematic Interventions to assess the quality of studies. It includes the evaluation of randomization methods, concealment of distribution, blindness of patients and physicians, outcome evaluation, data integrity, selective reporting, and other biased sources^26^.

### Statistical analysis

STATA (Version 17.0) command'mvmeta' was used to perform a multivariate network meta-analysis within a frequentist framework. The therapeutic effect of each study on NAFLD was calculated by standard mean deviation (SMD) and standard deviation (SD). For studies that provide only median and quartile ranges, we derive SMD and SD to overcome the heterogeneity between research interventions and results^27,28^. The I^2^ statistics are used to measure heterogeneity, which is considered high heterogeneity when it exceeds 50%. Consistency means that the treatment effect estimated by direct comparison is consistent with that estimated by indirect comparison. Statistical indicators include TG, TC, LDL-C, HDL-C, AST and ALT. Subsequently, interventions were ranked using the surface under the cumulative ranking curve (SUCRA). SUCRA is considered to be a more accurate estimate of the cumulative ranking probability. Simultaneously, SUCRA reported the overall probability based on the ranking of all interventions, that is, a given intervention is one of the best treatments^29,30^. When calculating the SUCRA impact of each exercise on NAFLD indicators, it is necessary to combine the corresponding indicators and observe the impact of exercise on the paired indicators.

## Result

### Literature selection

**Figure 1:**
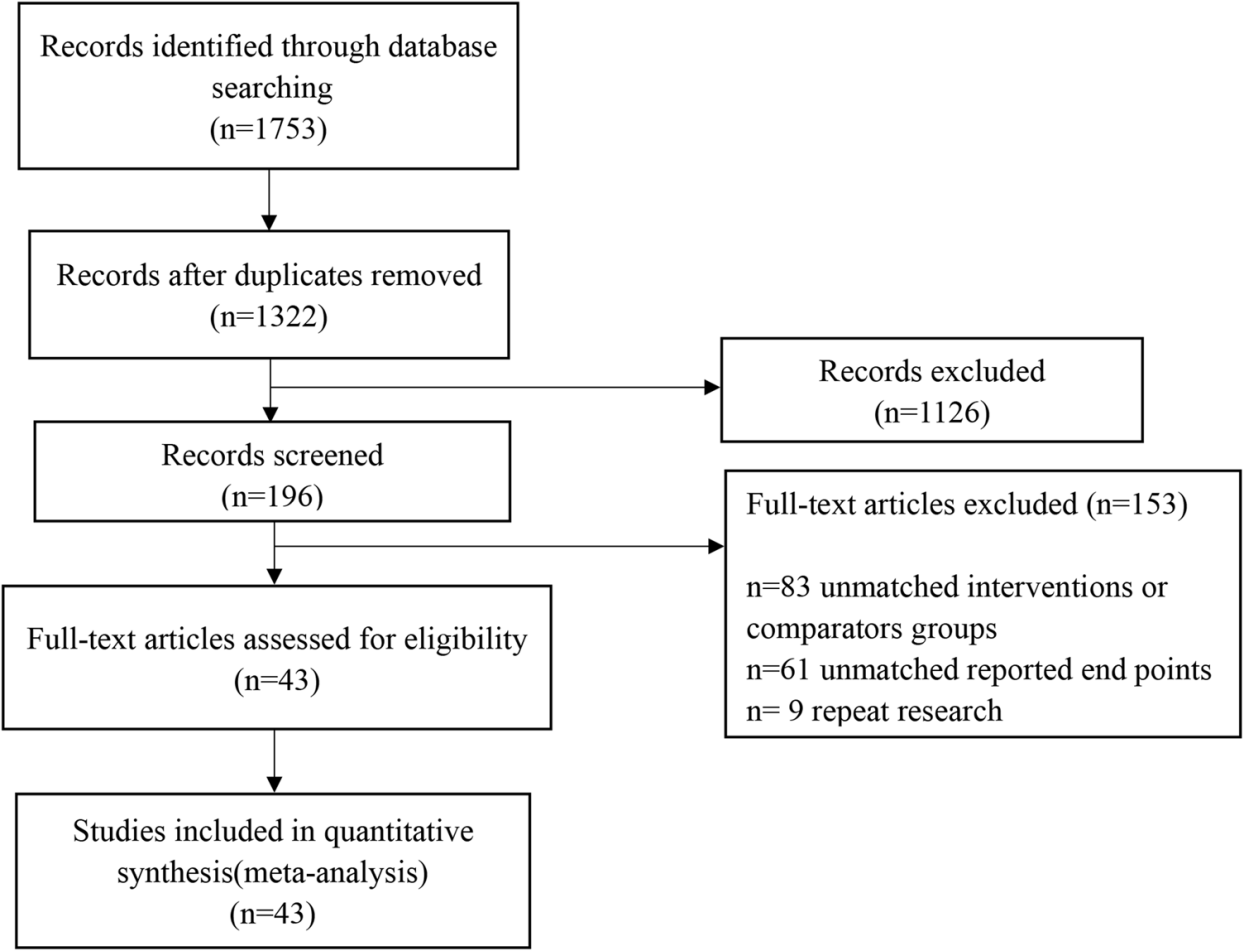
Study flow diagram.

A total of 1753 studies were initially identified. After reviewing the title, summary and full text, 43 studies met the inclusion criteria. Among them, there are 21 Chinese studies^31–51^ and 22 English studies^52–73^ in this review (Fig. 1).

### Characteristics of the included studies

The characteristics of these included studies are presented in Table1. A total of 2070 NAFLD patients were included in 42 studies, including 876 in the control group and 1194 in the intervention group. The exercise cycle varies from 8 to 24 weeks, and exercise modulations include aerobic exercise, resistance exercise, HIIT, aerobic exercise + resistance exercise. There is aerobic training (AT) (n = 779), resistance training (RT) (n = 159), high-intensity interval training (HIIT) (n = 160), aerobic training + resistance training (AT + RT) (n = 96) (Table 1).

**Table 1:** Basic characteristics of the included studies.

No.	Author	Sample size		Treatment		Interventiontime (weeks)	Outcome indicator	Other outcome
		T	C	T	C			
1	Hallsworth et al.^64^	11	8	RT	None	8	1, 5, 6	None
2	de Piano^66^	AT:15 RT:15	14	AT AT + RT	None	48	1, 2, 3, 4, 5, 6	None
3	Sullivan^53^	12	6	AT	None	16	1, 2, 5	None
4	Pugh et al.^56^	13	7	AT	None	16	1, 2, 3, 4, 5, 6	Decreased liver fat content
5	O Al-Jiffri^71^	50	50	AT	None	12	4, 5	None
6	Jakovljevic^61^	9	8	RT	None	8	1, 5, 6	None
7	Hallsworth^63^	12	11	HIIT	None	12	1, 4, 5, 6	None
8	Shamsoddini^55^	AT:10 RT:10	10	AT RT	None	8	4, 5	None
9	Keating^59^	36	12	AT	None	8	1, 2, 3, 4, 5, 6	None
10	Croci^68^	10	6	AT	None	24	2, 3, 6	None
11	de Lira^67^	HIIT:26 AT:25	33	HIIT AT	None	12	1, 2, 3, 4, 5, 6	None
12	Oh et al.^57^	AT:33 RT:19		AT RT		12	4, 5, 6	None
13	Winn et al.^88^	AT:5 HIIT:5	5	AT HIIT	None	4	1, 2, 3, 4, 5, 6	None
14	Keating et al.^60^	7	5	HIIT	None	12	1, 2, 3, 4, 5, 6	None
15	Stine et al.^89^	18	10	AT	None	20	1, 2, 3, 4, 5, 6	None
16	Charatcharoenwitthaya et al.^69^	AT:18 RT:17		AT RT		12	1, 2, 3, 4, 5, 6	None
17	Whyte et al.^52^	HIIT:16 AT:15	16	HIIT AT	None	8	1, 2, 3, 5, 6	None
18	Abdelbasset et al.^73^	15	12	AT	None	16	2	None
19	Moradi^58^	12	11	RT	None	12	4, 5	Structural changes in the liver
20	Ghamarchehreh^65^	AT:10 RT:10	8	AT RT	None	8	1, 2, 3, 6	None
21	Banitalebi et al.^70^	AT:17 AT + RT:17	18	AT AT + RT	None	10	4, 5	Fatty liver index decreased
22	Abdelbasset et al.^72^	16	16	HIIT	None	8	1, 2, 3, 5, 6	None
23	Peng^42^	27	27	AT	None	12	1, 2, 3, 4, 5, 6	Altered fatty deposits in the liver
23	Liu^45^	48	44	HIIT	None	12	1, 2, 3	None
25	Luo^44^	30	30	HIIT	None	12	1, 2, 3, 4, 5, 6	None
26	Zuo^31^	12	12	AT	None	24	1, 2, 3, 6	None
27	Liu^46^	30	30	AT	None	16	1, 2, 3, 5, 6	None
28	Fu^48^	AT:37 RT:37	36	AT RT	None	16	1, 2, 3, 6	None
29	Yang^37^	AT:34 RT:34	35	AT RT	None	20	1, 2, 3, 5, 6	None
30	Zhao^32^	17	14	AT	None	35	1, 3, 6	
31	Yang^36^	48	48	AT	None	24	1, 2, 3, 6	None
32	Yao^35^	15	15	AT	None	24	1, 2, 4, 5, 6	None
33	Xu^38^	42	29	AT	None	12	1, 2, 3, 6	None
34	Guo^47^	18	15	AT	None	24	1, 5, 6	None
35	Chen^50^	36	51	AT	None	52	1, 6	None
36	Mao^43^	30	30	AT	None	12	1, 2, 3, 6	None
37	Fan^49^	11	10	AT	None	16	2, 3, 5, 6	None
38	Zhang^34^	60	60	AT	None	16	1, 2, 3, 6	None
39	Wu^39^	15	15	AT	None	16	2, 3, 5, 6	None
40	Wu^40^	13	13	AT	None	16	2, 3, 6	None
41	Tan^41^	18	19	AT	None	24	1, 2, 3, 6	None
42	Zhang^33^	14	13	AT	None	24	1, 2, 3, 6	None
43	Li ying^51^	64	64	AT + RT	None	12	1, 2, 3, 6	None

1 TC 2 HDL-C 3 LDL-C 4 AST 5 ALT 6 TG.

*C* Control group; *T* Training group; *AT* Aerobic training; *RT* Resistance training; *HIIT*: High-intensity-interval-training; *AT + RT* Aerobic training + Resistance training.

### Result of assessment

**Figure 2:**
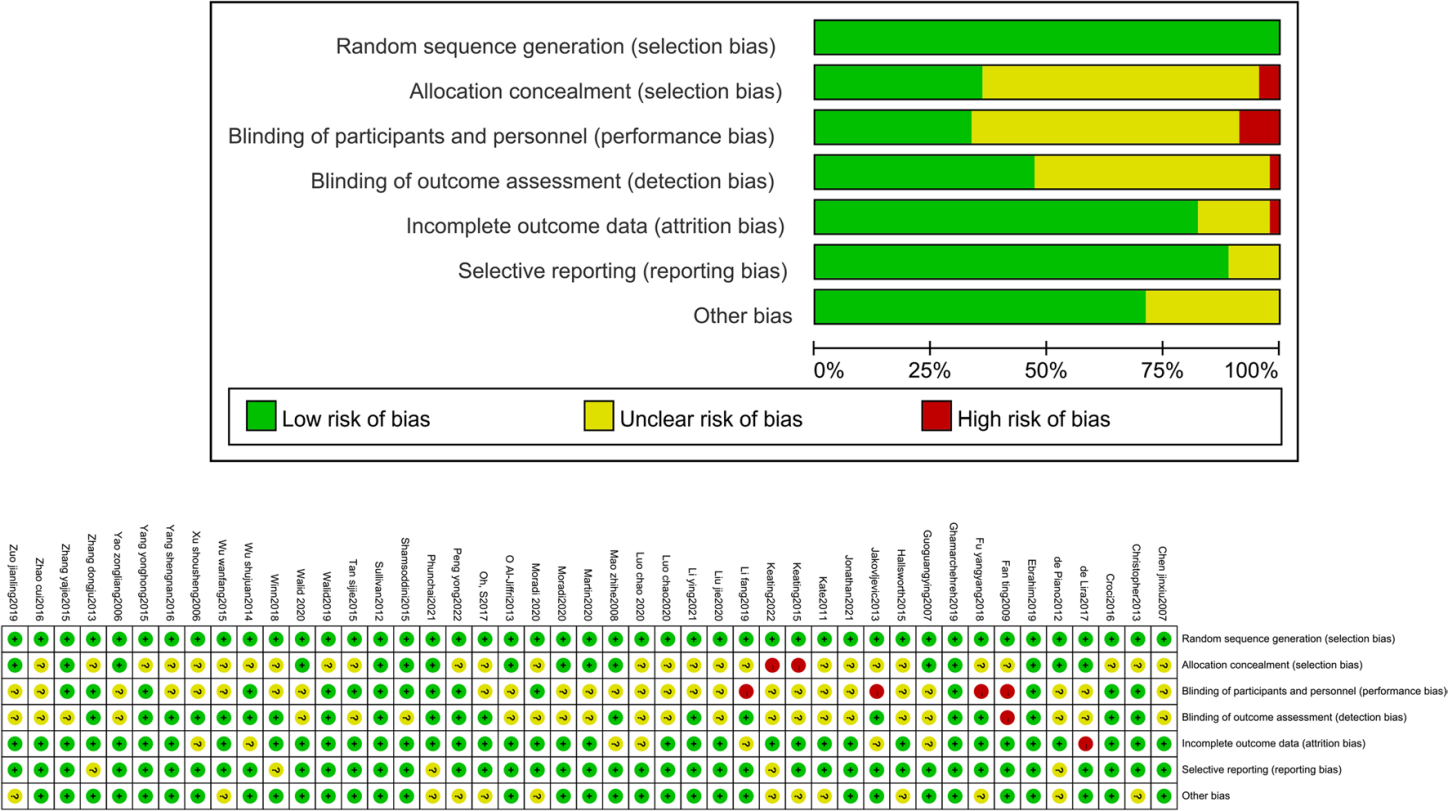
Schematic of cochrane bias risk assessment.

According to the Cochrane Intervention System Evaluation Manual, the quality of the study was assessed by the quality assessment method of the RCT. The Cochrane Bias Risk Assessment Chart shows the risks of different biases in 43 studies. In the included studies, the selection of new concepts to check whether blinding performance bias and outcome evaluation detection bias showed the highest risk. In addition, other biases such as attribution bias, reporting bias and random sequence generation show low risk (Fig. 2).

### Network meta-analysis

**Figure 3:**
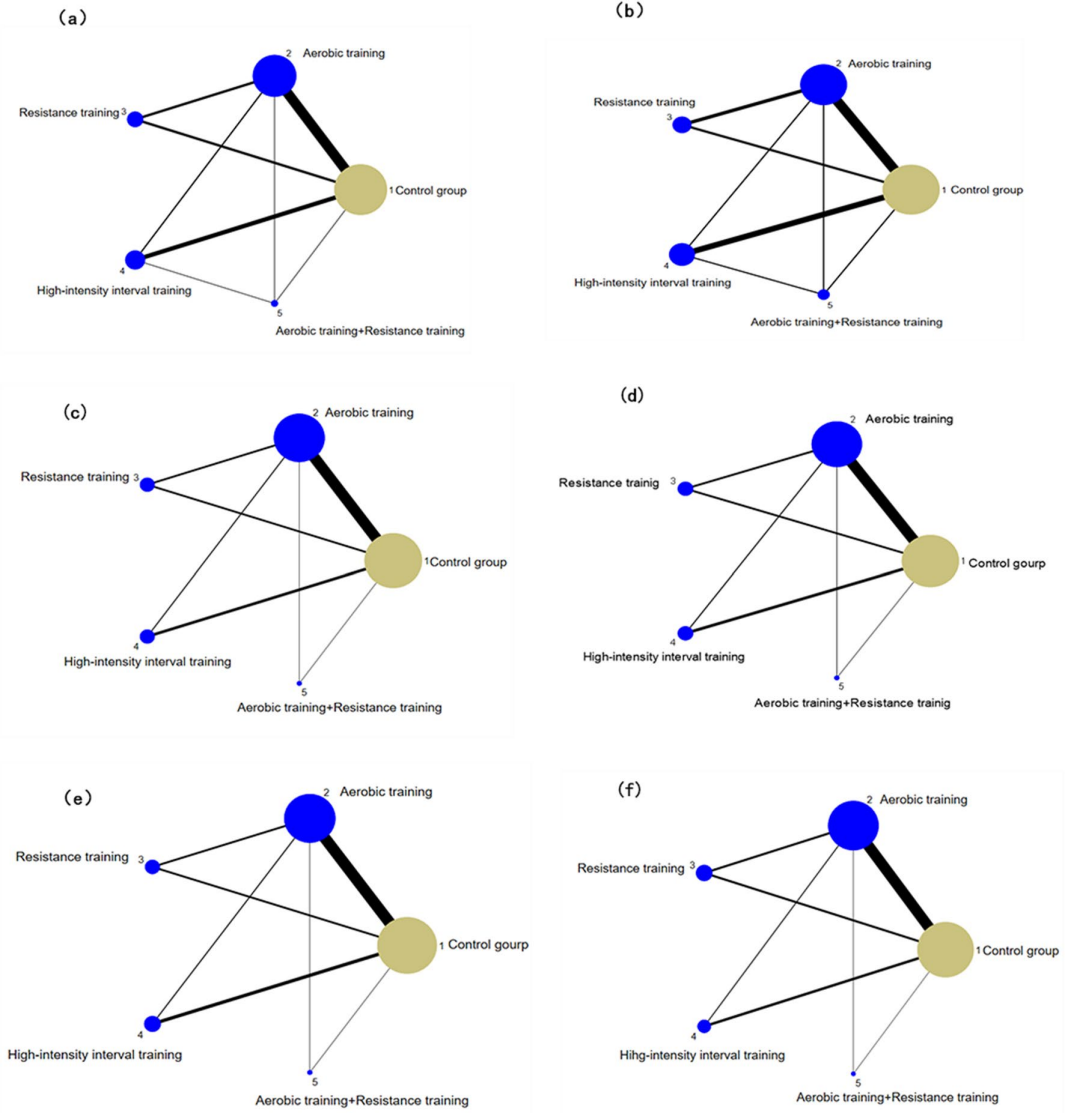
Effects of different exercise modes on lipids, liver enzyme networking, and plasma cholesterol in patients with nonalcoholic fatty liver disease. (**a**) ALT (**b**) AST (**c**) HDL-C (**d**) LCL-C (**e**) TC (**f**) TG.

This study includes various types of exercise therapy, HIIT, RT, AT, and AT + RT. The effects of four different exercises on TC, TG, AST, ALT, HDL-C and LDL-C in NAFLD patients were analyzed. Figure 3 shows the network meta-analysis of the effects of different exercise interventions on efficacy. The size of the node is related to the number of participants in the exercise interventions, and the thickness of the lines between different nodes is related to the number of studies compared. Compared with the control group, AT (*p* < 0.01), RT, HITT, AT + RT and other exercise modes can reduce the TC level of NAFLD patients. Similarly, for TG, AT (*p* < 0.01), HIIT (*p* < 0.05) and AT + RT (*p* < 0.01) show significant differences. Different exercise modalities can reduce HDL-C and ALT levels in NAFLD patients, and AT (*p* < 0.01) shows a significant decrease. The high expression of LDL-C in vivo brings bad benefits, which can be effectively reduced by different exercise modalities. On the contrary, RT (0.16 ( − 0.30, 0.62)) did not show a good reduction effect. For AST, RT (0.26 ( − 2.62, 3.14)) and AT (0.09 ( − 2.75, 2.93)) have no effect on AST reduction. About ALT, a significant improvement effect is brought by AT (*p* < 0.01). However, AT + RT does not appear to offer any significant improvement benefits (Table 2). Not only the global inconsistency, but also the node-splitting method is used to continue the local inconsistency inspection. Then, the node splitting method confirmed the difference in the influence of different motion modes on NAFLD patients. It was found that the improvement effect of AT VS RT (*p* < 0.05) on NAFLD was significantly different between TC, TG and LDL-C (Table 3). Subsequently, we will calculate the effect of different exercise modes on NAFLD patients through SUCRA.

**Table 2:** Global inconsistency detection.

Non-conformance inspection				
(vs Control)	n	95%CI		*p*
TC				
AT	25		− 0.69 ( − 1.11, − 0.26)	0.001*
RT	5		− 0.41 ( − 1.45, 0.63)	0.438
HIIT	7		− 0.61 ( − 1.86, 0.64)	0.339
AT + RT	2		− 1.16 ( − 2.86, 0.533)	0.179
TG				
AT	28		− 0.58 ( − 0.91, − 0.25)	0.001*
RT	7		− 0.23 ( − 1.08, 0.61)	0.59
HIIT	6		− 1.10 ( − 2.16, − 0.37)	0.043*
AT + RT	2		− 1.98 ( − 3.37, − 0.59)	0.005*
HDL-C				
AT	25		0.61 (0.09, 1.12)	0.022*
RT	5		0.48 ( − 0.73, 1.69)	0.434
HIIT	6		0.61 ( − 0.87, 2.01)	0.419
AT + RT	2		1.22 ( − 0.81, 3.26)	0.237
LDL-C				
AT	23		− 0.56 ( − 0.77, − 0.34)	0.00*
RT	5		0.16 ( − 0.30, 0.62)	0.497
HIIT	6		− 0.99 ( − 1.58, − 0.40)	0.001*
AT + RT	2		− 1.01 ( − 1.69, − 0.35)	0.003*
AST				
AT	11		− 0.66 ( − 1.83, 0.50)	0.264
RT	4		0.26 ( − 2.62, 3.14)	0.860
HIIT	5		− 0.40 ( − 3.20, 2.39)	0.777
AT + RT	2		0.09 ( − 2.75, 2.93)	0.950
ALT				
AT	19		− 0.85 ( − 1.26, − 0.43)	0.00*
RT	6		− 0.14 ( − 1.13, 0.84)	0.773
HIIT	7		− 0.73 ( − 1.70, 0.24)	0.140
AT + RT	2		0.56 ( − 1.37, 1.49)	0.938

*AT* aerobic training; *RT* resistance training; *HIIT* high intensity interval training; *AT + RT* aerobic training + resistance training; *p < 0.05.

**Table 3:** Node-splitting inconsistency detection.

Group	Direct	Indirect	*p*
TC			
AT VS RT	− 1.03	1.42	0.002*
AT VS HIIT	− 0.17	0.67	0.721
AT VS AT + RT	− 0.32	0.96	0.772
TG			
AT VS RT	0.23	1.45	0.048*
AT VS HIIT	− 0.17	− 0.18	0.996
AT VS AT + RT	− 0.44	− 1.45	0.336
HDL-C			
AT VS RT	− 0.10	− 0.08	0.986
AT VS HIIT	0.26	− 0.77	0.207
AT VS AT + RT	− 0.58	0.66	0.363
LDL-C			
AT VS RT	− 0.26	1.36	0.000*
AT VS HIIT	− 0.91	− 0.40	0.510
AT VS AT + RT	− 0.36	− 0.58	0.450
AST			
AT VS RT	− 0.82	0.19	0.485
AT VS HIIT	0.31	0.27	0.975
AT VS AT + RT	− 0.60	0.37	0.583
HIIT VS AT + RT	− 0.22	− 0.53	0.868
ALT			
AT VS RT	− 0.78	0.57	0.083
AT VS HIIT	0.07	0.09	0.971
AT VS AT + RT	− 1.01	0.50	0.226
HIIT VS AT + RT	0.12	− 0.64	0.569

*AT* aerobic training; *RT* resistance training; *HIIT* high intensity interval training; *AT + RT* aerobic training + resistance training; *p < 0.05.

### The effect of different exercise modalities

#### TC and TG

**Figure 4:**
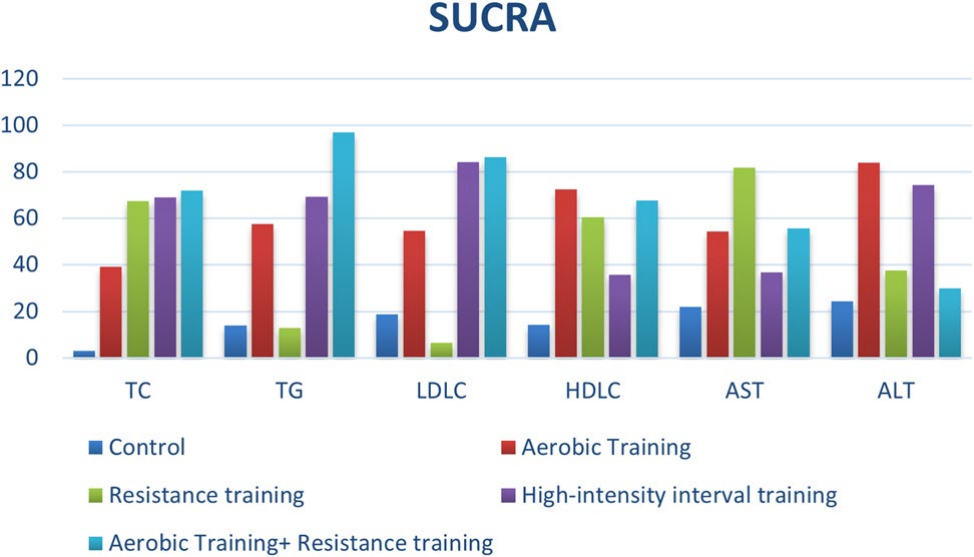
Ranking chart of different outcome indicators for each intervention.

**Figure 5:**
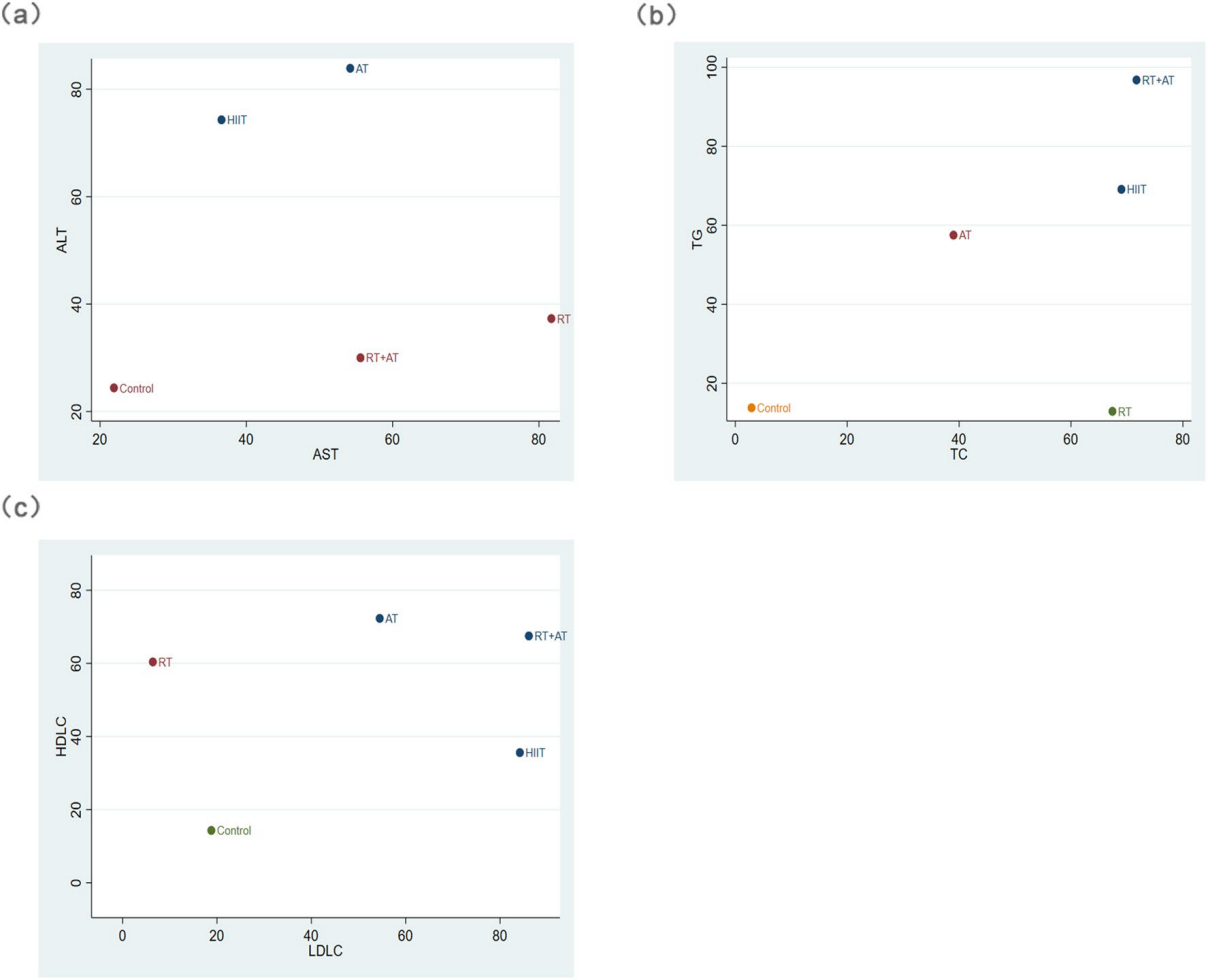
Efficacy of different exercise modalities. (**a**) ALT and AST (**b**) TG and TC (**c**) HDL-C and LDL-C.

In the network analysis, SUCRA is considered to be a more accurate estimate of the cumulative ranking probability. It can use different ranking methods, maximum or minimum, according to the different benefits of sports. Among the effects of different exercise modes on TC in NAFLD patients, AT + RT (SUCRA = 71.7) has the best effect, HIIT (SUCRA = 69.0), RT (SUCRA = 67.4), AT (SUCRA = 39.0). For TG, RT + AT (SUCRA = 96.8) still provides the best intervention benefits. HIIT (SUCRA = 69.1), AT (SUCRA = 57.5), RT (SUCRA = 12.9) (Fig. 4). Subsequently, after fitting the effects of different exercise modes on TC and TG, it was found that AT + RT had the best overall effect on TC and TG reduction in NAFLD patients, followed by HIIT (Fig. 5a). HIIT also takes great improvement.

#### ALT and AST

ALT and AST are two important indicators of liver function. By calculating the effect of different exercise modalities on ALT, AT (SUCRA = 83.9) had the best effect on ALT in NAFLD patients. HIIT (SUCRA = 74.3), RT (SUCRA = 37.3), RT + AT (SUCRA = 30). For AST, RT (SUCRA = 81.7), RT + AT (SUCRA = 55.6), AT (SUCRA = 54.2), HIIT (SUCRA = 36.6) (Fig. 4). Among them, AT has the best effect on reducing ALT and AST in patients with NAFLD (Fig. 5b).

#### LDL-C and HDL-C

LDL-C is a kind of bad cholesterol, which is usually too high in patients with NAFLD. By calculating the effect of different exercise modalities on LDL-C, RT + AT (SUCRA = 86.1) had the best effect on LDL-C in NAFLD patients. HIIT (SUCRA = 84.2), AT (SUCRA = 54.5), RT (SUCRA = 6.4). For HDL-C, AT (SUCRA = 72.3) is the best exercise modality in NAFLD patients, subsequently, RT + AT (SUCRA = 67.5), RT (SUCRA = 60.4), HIIT (SUCRA = 35.6) (Fig. 4). Through comprehensive effect verification, AT + RT is the best exercise modalities, which can improve HDL-C and reduce LDL-C in NAFLD patients. AT also brought great improvement (Fig. 5c).

## Discussion

Exercise brings many benefits to NAFLD patients, such as promoting blood lipid metabolism, reducing liver fat, and improving quality of life, etc. Many studies have shown that exercise intervention is effective for NAFLD patients^22,74,75^. However, considering individual heterogeneity, it remains a challenge to develop appropriate exercise prescriptions for NAFLD patients. Therefore, it is of great significance to explore the best exercise mode for NAFLD patients to improve the exercise intervention and improve the symptoms of NAFLD patients. Our study found that AT + RT is the best for overall improvement of TC and TG in NAFLD patients. The results are consistent with previous studies^76^. For ALT and AST, we found that the best exercise modality to improve them is AT and RT, respectively. AT has the best effect on ALT levels in NAFLD patients, while RT has the best effect on AST levels in NAFLD patients. This is not consistent with the AT + RT obtained by ZHOU et al.^76^, which may be due to the fact that we included more RCTs than they did. In addition, we also added the effect of exercise on LDL-C and HDL-C, which are important for improving NAFLD patients. RT + AT and AT are the best exercise methods to improve LDL-C and HDL-C in NAFLD patients. In terms of overall effect, our conclusion is that AT + RT is the best exercise method to improve NAFLD patients.

AT + RT exercise mode is the best exercise mode we have found. It is a combination of AT and RT. The test indicators we selected include TG, TC, AST, ALT, LDL-C, HDL-C. Because TC is an independent factor in the development of cirrhosis, TG is an independent predictor of cardiovascular disease^77,78^. High levels of AST and ALT increase the risk of liver cell damage^79^. These two liver enzymes are also key parameters for determining NAFLD and hepatitis incidence. HDL-C is mainly synthesized in the liver and is an anti-atherosclerotic lipoprotein, which can transport cholesterol from extrahepatic tissues to the liver for metabolism and excrete it from bile. However, some studies have found that HDL-C levels are reduced in NAFLD patients^80^. LDL-C is the main lipoprotein in fasting plasma and the main vehicle for transporting cholesterol to extrahepatic tissues. Some studies have found that almost one in 10 children and adolescents with NAFLD have low LDL-C levels, which is about twice the expected level of the general population. Interestingly, patients with NAFLD and low LDL-C levels have similar liver disease severity as patients with normal or elevated LDL-C^81^. One characteristic of movement is that it consumes a lot of energy in the process. Long-term exercise significantly improves ALT and AST in Chinese patients with NAFLD^82^. Elevated transaminase levels are considered an independent predictor of advanced fibrosis, and have also been shown to correlate significantly with NASH^83,84^. And the ratio of AST to ALT is often used in medicine and is considered an independent indicator for predicting advanced liver fibrosis^85^. Improvements in this ratio from exercise can reflect the benefits that exercise brings to NAFLD patients. Exercise is beneficial for reducing visceral fat. A 4-week AT intervention experiment was conducted on 19 sedentary obese people. It has been found that AT can reduce visceral adipose tissue volume by 12% and liver fat content by 21% over a 4-week period. High intensity physical activity has been reported to effectively improve the pathological conditions of NAFLD, including fat accumulation, inflammation and fibrosis^57^. This strongly confirmed the benefits of physical exercise for improving NAFLD patients. However, in exploring which exercise can achieve the best effect, some studies have confirmed the benefits of combined exercise (AT + RT) through experiments^86^. Among obese adolescents diagnosed with metabolic syndrome, AT + RT is more effective than AT alone in improving the associated inflammatory process, including increasing adiponectin concentration and controlling cardiovascular risk factors^87^. One study found that the cure rate of AT + RT combined training for NAFLD patients was higher than that of AT alone after 60 patients received AT alone or AT + RT combined training for one year^66^. Regarding the exercise cycle, the research intervention duration we included is generally 12 weeks. Perhaps the effects of exercise need to be achieved over a long period of time. Combined training (AT + RT) was more effective than AT alone in promoting high amplitude changes in fat mass (kg), lean mass (kg, %), homeostasis model assessment-Insulin resistance (HOMA-IR), LDL-C, adiponectin, adiponectin/leptin ratio, and mean corpuscular hemoglobin (MCH)^66^. Most patients with NAFLD are middle-aged or older, and attention should be paid to the patient's physical condition when choosing exercise methods. Therefore, finding more scientific training methods is of great significance for the rehabilitation of NAFLD patients.

Although this NMA has many benefits, it must admit some limitations: (1) There is a large difference in the number of exercise modes included in each study; (2) Only the choice of motion mode is considered, and the choice of motion content, period and frequency is ignored; (3) The article only includes Chinese and English, and there are high-quality RCTs in other languages that are not included; (4) Only assessed changes in blood biomarkers, and lacked more intuitive indicators associated with NAFLD, including steatosis inside the liver, biopsy, ultrasound or elastography tests.

## Conclusion

Through network meta-analysis, we found that for patients with NAFLD, AT + RT modalities is the best way to improve TC, TG and LDL-C. Next, AT is the best mode to improve ALT and HDL-C. RT is best mode to improve AST. For the overall effect, AT + RT has the best effect on improving TC, TG, LDL-C and HDL-C in NAFLD patients. AT has the best effect on improving AST and ALT in patients with NAFLD. Therefore, NAFLD patients are recommended to participate in AT OR AT + RT modes improving NAFLD. In addition, we also added the effect of exercise on LDL-C and HDL-C, which are important for improving NAFLD patients. Therefore, our results show that AT + RT at least three times a week, 50 min each time, for 10 weeks, has a better effect on liver improvement in NAFLD patients.

## Data Availability

All data generated or analysed during this study are included in this published article.

## Abbreviations

NAFLD: Nonalcoholic fatty liver disease
NMA: Network meta-analysis
T2DM: Type 2 diabetes mellitus
ROS: Reactive oxygen species
OS: Oxygen species
TC: Triglycerides cholesterol
TG: Triglyceride
LDL-C: Low-density lipoprotein cholesterol
HDL-C: High-density lipoprotein cholesterol
AST: Aspartate transaminase
ALT: Alanine aminotransferase
RCTs: Randomized clinical trials
PRISMA: Preferred reporting items in the system review and meta-analysis
SMD: Standard mean deviation
SD: Standard deviation
SUCRA: Surface under the cumulative ranking curve
C: Control group
T: Training group
AT: Aerobic training
RT: Resistance training
HIIT: High-intensity-interval-training
AT + RT: Aerobic training + Resistance training
HOMA-IR: Homeostasis model assessment-insulin resistance
MCH: Mean corpuscular hemoglobin
